# Formation of an Anti-Chinese Consensus among US “Think Tanks”: From D. Trump to J. Biden

**DOI:** 10.1134/S1019331622130044

**Published:** 2022-12-23

**Authors:** D. A. Kochegurov

**Affiliations:** Arbatov Institute for US and Canadian Studies, Russian Academy of Sciences, Moscow, Russia

**Keywords:** foreign policy, United States, China, competition, consensus, world order, think tanks, restraint, confrontation

## Abstract

This article is dedicated to the factor of think tanks in the US foreign policy towards China. A rapid change in attitudes to China within the US political elite is recorded, which indirectly reflects the influence of think tanks on it. Based on the analysis of reports of leading think tanks, the evolution of their views on China is demonstrated. The main conclusion is that the think tanks support an anti-Chinese consensus, strengthened during the presidency of D. Trump, and advocate stricter measures against China. Overall, the American expert community sticks to the same approaches to China and gives approximately similar recommendations to the US government.

## INTRODUCTION

There is every reason to believe that China has come to be perceived by American elites as a direct and primary threat to US national interests. The American political establishment has abandoned the premise that interaction with competitors and their inclusion in international institutions and global trade will inevitably turn them into bona fide players and reliable partners. The old paradigm has been replaced by a new consensus, backed by the presidential administration, both parties, the military establishment, think tanks, and the mainstream media, which suggests that China is a threat to the United States, that the US China policy has failed, and that Washington needs a new, tougher containment strategy for Beijing.

The 2017 National Security Strategy (NSS), which has become an expression of “principled realism” with an emphasis on national interests, declared China a revisionist force that seeks to supplant the United States in the Indo–Pacific region (IPR), impose its economic model on others, and change the regional order in its favor [1]. No previous NSS used such categorical, confrontational language to describe Beijing’s behavior in the world and towards the United States. The 2018 National Defense Strategy (NDS) highlighted China as a strategic competitor and noted that, as China continues its economic growth, it will develop a military modernization program aimed at asserting its hegemony in the IPR and achieving global dominance [2].

In the very first doctrinal document of the Biden administration, Interim National Security Strategic Guidance, published in March 2021, J. Biden admitted that the balance of power in the world had changed unfavorably for the United States, and the world simply cannot return to its previous state; therefore, the United States must develop a new course in its foreign and domestic policy [3]. As in the 2017 National Security Strategy, the return to great-power rivalry, which had acquired a strategic character, was stated. More assertively, China was hailed as the main geopolitical challenge to a stable and open international system, while issues of interaction and cooperation with it enjoyed little attention. The National Defense Strategy 2022 declares China the number one challenge, and its containment in the IPR, the main strategic priority [4].

In this respect, both the Trump administration’s doctrinal documents and the White House’s March document, which anticipated the imminent emergence of the National Security Council 2022, largely echo the reports of the leading American think tanks that influence the formation and implementation of Washington’s real foreign policy. Trump’s 2017 setting, adopted by Biden, that the main content of world politics was the resumption of geopolitical rivalry between great powers, among which China is the main threat to the United States, was borrowed, among other things, from the positions of foreign policy experts stated earlier, which requires close analysis.

## THE LEADING US THINK TANKS: COMPARING THEIR APPROACHES TO CHINA

Over a hundred years, a wide network of independent and nonpartisan organizations has developed in the United States, which are responsible for identifying and solving objective national problems, determined with account for the structure of the international system and its own domestic political situation. These organizations are defined as *think tanks* or *brain centers*, which have concentrated the scientific and intellectual potential of the United States. Leading think tanks have become an integral part of the US political system, including its foreign policy machinery. Think tanks, considering their influence, are ranked as the “fifth power.” Their main foreign policy function is to conduct research commissioned by government departments or congressional committees to revise policies in relation to a particular region or countries, or to reform a specific segment of the US foreign policy mechanism [Samuylov, 2013, p. 284].

A lot of fundamental scientific works by S.M. Samuylov, V.B. Supyan, and other scientists have been dedicated to the topic of think tanks. We will focus on the position of the leading American think tanks in relation to China, relying on their hierarchy in descending order in terms of their influence in the world. In ranking them, we proceed from the authoritative world ranking *2020 Global Go to Think Tank Index Report*, compiled by the University of Pennsylvania at the request of the UN [5]. An obligatory criterion for its formation is the participation of a think tank in applied analytics that has a significant impact on the ongoing state policy.

*The Brookings Institution* is a center–left think tank founded in 1916. In February 2020, Brookings Institution Vice President B. Jones noted that, even at the time of deep internal division in the United States, there is an elite and social consensus on China with account for “Beijing’s shift in strategy towards a more assertive posture towards the West” [6, p. 1]. China has abandoned its “peaceful rise” in favor of an assertive, nationalistic, and ideological approach focused on weakening the influence of the United States in the world, and therefore the US response, according to Jones, should be to strengthen alliances and protect the basic principles of the liberal world order with the mobilization of all available resources [6, p. 4]. Reproaching the Trump administration for “myopic underattention” to alliances and multilateralism, Jones emphasized that, for the first time in 200 years, an illiberal authoritarian regime was ready to play an important role in writing the rules of the world order; however, the Chinese model of development is unacceptable to the West, and therefore the world stands on the threshold of a bifurcation of globalization and the emergence of two competitive zones [6, p. 6].

Other analysts also noted in February 2019 that the US−China relationship (especially trade and economic) had reached a breaking point. In their opinion, US concerns underlying bilateral tensions stem from specific practices inherent in the Chinese economic model; the American knowledge economy is under attack, including high-paying jobs and high value-added industries [7, p. 2]. China’s industrial policy, which distorts the rules of the game in its own favor, contradicts the market systems of most WTO member countries [7, p. 3]. Therefore, the US strategy, the institution’s analysts say, should include raising trading standards through new free trade zone agreements (FTAs), whose participants will benefit from them and “create economic costs to China,” which should encourage it to reform its economy. The outcome of the competition with China will ultimately be determined by the actions taken by the United States at home. In addition to increasing competitiveness, it is necessary to control access to technologies, as well as effectively use the tariffs agreed with the WTO, minimizing the damage to business [7, p. 5]. The progress that the United States already made with the Foreign Investment Risk Review Modernization Act (FIRRMA) of 2018 and the Export Control Reform Act (ECRA) was noted.

In November 2020, a fundamental work was released containing recommendations for the Biden administration on China [8]. Responding to the Chinese challenge will require the United States taking four important steps: strengthening the economy through reform and investment, engaging with allies to push China to open its economy and develop 21st century trade rules, strengthening the military presence in the region, and working with China on issues of common interest. The authors of the report acknowledged that the United States should abandon the idea of changing the Chinese political regime and preventing the rise of China through unilateral steps. It was recommended adapting to modern China and the challenges it poses to US interests and values. Trump’s unilateral approach was not a success in containing China. The United States, according to the authors of the report, needs to develop a new strategy that includes strengthening economic competitiveness, increasing confidence in US security commitments, and protecting American values. The United States is engaged in a long-term systemic competition with China. The military−industrial complex (MIC) will play an important but not central role in measuring progress in this rivalry. Rather, the outcome will depend on whose governance model is more attractive for improving the lives of citizens and solving the key problems facing the world [9].

*The Carnegie Endowment for International Peace* is a center-left think tank founded in 1910.

Standing out among the works of the foundation devoted to the US–China rivalry are the reports of A. Tellis, an analyst and ideologist of the US–India rapprochement, compiled by him and his colleagues from the National Bureau of Asian Research. Back in January 2014, Tellis noted that the intensive development of China was the most serious geopolitical problem for the United States and that the challenge thrown out by China would be more serious than the challenge from the Soviet Union [10, p. 4].

In January 2020, a report was published in which Tellis explains that Trump only formulated what the two previous administrations had assumed as a likely scenario but were afraid to admit openly. However, Trump was late with taking a number of measures: the projection of might in Asia, the creation of the Indo–Pacific coalition, and the preservation of the technological dominance of the United States [11, p. 39]. The report questioned the effectiveness of tariffs as a means of reducing the negative balance of trade but supported their use as a tool for forcing China to remove trade barriers [11, p. 41]. The main thing is that Washington should adjust its policy that will allow it to compete more successfully [11, p. 43]. Thus, the United States needs to invest in itself while remaining a global source of innovation. In coordination with its allies, it should work on reforming the trading system. The time has come to sign new plurilateral free trade agreements that would bind the United States more closely to its partners and limit China’s access to advanced technologies. Finally, it is important to restore the ability of the United States to project power in the region. If the United States wants to maintain its primacy in the face of increasing competition from China, it should behave like a responsible power.

According to the foundation’s analysts, President Biden largely adheres to the policy of Trump. Two distinct elements of continuity show this. First, the Biden administration accepted as a reality the fact that China is a strategic competitor and rival of the United States. Second, his foreign economic team did not depart from the principles of mercantilism and protectionism in international trade and accelerated the Buy American and Hire American program [12, p. 34].

*The Center for Strategic and International Studies (CSIS)* is a center-right think tank founded in 1962. In January 2018, Chief Executive Officer of the center J. Hamre recorded that the ambivalence in US−China relations had come to an end. The most important element of Beijing’s “revanchism” and “nationalist” strategy is to change global rules and institutions in accordance with its interests and bypass the United States [13, p. VI]. More confident and assertive, China will not change course without external pressure, which will not materialize without American leadership [13, p. VII]. There is a consensus in the United States that China’s industrial policy is contrary to American interests. There is a consensus on the need to take measures using export controls, trade duties, and restrictions on foreign investment [13, p. VIII]. According to Hamre, the optimal strategy should include three elements: targeted investments in infrastructure, research, and new technologies; their combination with regulatory instruments; and exerting pressure on China that would force it to change its behavior [13, p. XI].

In September 2019, a group of scientists established that the trade and economic conflict between the United States and China was likely to become a characteristic feature of bilateral relations for many years and could result in a partial separation of the two economies [14, p. 1]. Five recommendations were made: establishing a “dual credibility,” in which Washington must convince Beijing that it is ready to impose tariffs and bear the costs associated with this and at the same time meet its obligations if a mutually beneficial deal is reached; setting clear goals while assessing costs and benefits; improving the decision-making process; creating a multilateral coalition, when the mobilization of allies and partners can play on Beijing’s fear of isolation; and investing in its own economy as the basis of competitiveness on the world stage [14, p. 7].

In May 2020, the expert A. Cordesman also noted increased competition from China. He determined that, where possible, China will use its power, initiating a “war of influence” in ways that do not involve real hostilities. To combat this more effectively, the United States must rethink its military strategy and forces, with a focus on gray areas and hybrid conflicts. It should reorient itself to respond better to global challenges at the national and regional levels and to combine its military, political, and economic strategies [15, p. 3]. In January 2021, Cordesman repeated that it was necessary to make changes to the model of competition with China; if the United States wants to develop a more effective approach, it should look at it differently than just as an arms race [16, p. 2].

*The RAND Corporation* is a center-right think tank established within the US Air Force in 1948. A 2020 RAND report confirmed that the United States and China had entered an era of great power rivalry, part of which is not only military and economic confrontation but also the struggle of two ideologies [17]. In a situation when ideological differences reinforce the perception of threat, the states tend to perceive each other’s actions as more threatening. China, like Russia, has begun to form its own ideological project, challenging key aspects of the Western-centric world order. This is explained by the fact that as states become more powerful, their ideological ambitions tend to grow. Characteristics are a tendency to externalize internal forms of government and an attempt to reproduce oneself on the world stage.

In the June 2021 report, RAND Lead Analyst T. Heath emphasized that for the first time since confronting the Soviet Union in the Cold War, the United States faced the prospect of long-term great power rivalry. Note that Russia was characterized as a “rogue state,” capable of inflicting irreparable damage but not seriously challenging the status of the United States as a global leader [18, p. 3].

In December 2021, a report was released containing the recommendations of the Biden administration regarding the containment of China. Among other things, it discussed the following: the accumulation of elements of US national power to achieve an international consensus on China; supply chain diversification; investment in the military−industrial complex; joining new trade alliances; dominance in cyberspace; combating unfair economic practices that give China an advantage; promotion of an attractive image of the United States; investment in countries most vulnerable to China; creating partnerships, coalitions, and mechanisms for cooperation in the field of security, alternative to China; and fight against Chinese propaganda [19].

*The Center for American Progress (CAP*) is a progressive think tank founded in 2003. In April 2019, its experts noted that the main geopolitical challenge of the 21st century would be how the United States and the rest of the world would respond to the rise of China. If the Chinese vision of the world order prevails and it becomes the dominant power, there is a risk that the world will become less free, prosperous, and secure. From their point of view, Trump’s approach to China had two fundamental shortcomings: in economic terms, it does not create conditions for effective US competition, and politically, the United States has abandoned the role of world leader, alienating potential allies and partners who share similar concerns about China instead of working with them. If the United States stays on its current course, it will lose ground to China. To reverse this negative momentum, the United States must invest in its unique strengths: in domestic policy, to address economic problems and invest in factors of economic prosperity and national security; on a global scale, to return to a multilateral approach, to build and lead a single anti-Chinese bloc. Subsequently, the United States will be able to implement a strategy that will limit China’s room for maneuvering, encourage China to realize its potential for the benefit of the global common good, and allow the United States to compete in the long term [20].

*The Heritage Foundation* is a conservative think tank founded in 1973. In February 2020, the foundation’s leading analysts produced a special report providing insight into the factors shaping China’s behavior on the world stage. The report included more than 50 recommendations to help address Beijing’s growing influence. It noted the foundation’s utmost respect for the Chinese people; the problem is not with it but with the “communist dictatorship” that threatens the well-being of peoples around the world. The analysts welcomed the attention of politicians to the full range of threats emanating from China and recognized the need to solve them [21].

The main conclusion reached in April 2020 by Director of the Foundation’s Center for Asian Studies W. Lohman and Vice President J. Carafano is that great power rivalry with China is a long-term challenge to American national interests that has no clear historical analogy [22]. Ten steps were proposed to answer it: investigating the origin of the coronavirus COVID-19, conclusion of new FTAs, military buildup in the IPR, preparing the US economy for long-term competition, prohibition of Huawei and ZTE to participate in the development of the 5G network, the weakening of Chinese influence in international organizations, coordination of actions on export control and investment regimes with the European Union, countering economic espionage and technology theft, support for Taiwan, and upholding American values.

In general, reading the foundation’s analysts produces the impression that their position on China is categorical. The United States−China relations are considered in the context of opposition to one another. The page for the China section explains that China’s rise is a constant and formidable challenge that the United States will have to face over the next few decades; even before the COVID-19 pandemic, China was an “irresponsible global actor” that threatened American interests and values [23]. It is worth mentioning some notes from recent reports:

—Extreme concern about China’s expansionist activity in the light of its conclusion of a defense agreement with the Solomon Islands, which was the culmination of China’s efforts to break through the island lines of the United States and its allies and enter the Pacific Ocean (B. Sadler, April 2022).

—Support for the increased role of the Congress, ready for a large-scale debate on China. The US−China Economic and Security Review Commission and the Congressional-Executive Commission on China are think tanks that have already submitted 14 Priorities for a Comprehensive China Bill and Seven Principles for a Congress-Led China Policy (W. Lohman, April 2021).

—Recognition of the fact that China’s actions are ideologically motivated. The revival of ideology as a factor will affect China’s foreign and defense policies. The current ideology, rooted in Marxism–Leninism, Maoism, Chinese history, and Xi Jinping’s ideas, is fundamentally incompatible with the US ideology, and therefore, from the CCP’s point of view, the United States is an existential ideological threat (D. Cheng and O. Enos, March 2021).

—A new perception of Japan with support for strengthening the US−Japan alliance amid Tokyo’s hesitation in terms of abandoning post-war restrictions and taking greater responsibility for its security (B. Klingner, September 2020).

—The evolution of the Taiwanese factor, the cornerstone of China’s Indo–Pacific containment belt. The White House and Congress reached a consensus on strong support for Taiwan, and a bilateral FTA should be the next logical step in enhancing mutual trust and expanding economic engagement (A. Kim and W. Lohman, August 2020).

*The Council on Foreign Relations (CFR)* is a centrist think tank founded in 1921. In January 2020, a report by CFR expert R. Blackwill was released, which stated that, even if partial normalization is achieved in US−China relations, each side of the conflict will continue to view the other as a strategic adversary. The two countries have different histories, different values and political cultures, vital national interests, long-term foreign policy goals, and visions of the domestic and international order; therefore, they are unlikely to achieve a sustainable and stable balance in the near future [24, p. 2]. The US efforts to integrate China into the liberal world order have created threats to American leadership, so the United States needs a new big strategy towards China [24, p. 9]. The lengthy report presented 22 prescriptions stemming from the understanding that maintaining the central role of the United States in shaping the global system remains a major goal in the 21st century. The United States needs the following: to revive the economy, to create new preferential trade agreements using instruments that could remove China from the equation, to restore the technology transfer control regime, to build up military infrastructure on the periphery of China, to strengthen the US armed forces for their effective and rapid projection on the territory of Asia and vital sea routes, and to promote American values in the world [24, p. 10].

Blackwill admitted that, under the Trump administration, US−China relations had entered the fourth phase, the phase of competition, replacing the postwar confrontation, the thaw in relations under R. Nixon, the inclusion of Beijing in the international system with the hope that it would become a “responsible actor” and accept the rules of the liberal world order [25, p. 16]. Blackwill criticizes previous administrations for the fact that, long before Trump, they constantly talked about a strategic partnership with China and used false approaches, misinterpreting its true intentions. While American leaders were making optimistic statements, China was implementing its grand strategy to undermine the US position in the APR. Blackwill puts this miscalculation among the main foreign policy mistakes made after the Second World War, along with the decisions of 1965 and 2003 [25, p. 9]. Trump started with the questionable decision to withdraw from the Trans-Pacific Partnership (TPP), but since then a much clearer approach to China has been developed, breaking with many mistakes of the past, and therefore, in general, his presidency deserves high praise [25, p. 10].

In September 2019, with the participation of the cybersecurity expert A. Segal, a report was released stating that although Beijing’s attempts to become a technological power contributed to global growth and prosperity, and the United States and China benefited from bilateral trade and investment, China’s intellectual property theft and industrial policies posed a threat to the economic competitiveness and national security of the United States [26, p. 36]. China is implementing three industrial strategies: Guideline for the Promotion of the Development of the National Integrated Circuit Industry 2014, Made in China 2025, and Next Generation Artificial Intelligence Development Plan, which aim to make Chinese firms produce 70% of chips, upgrade the aging manufacturing base, and compete with the United States in the field of artificial intelligence (AI) [26, p. 40]. In response, the United States needs to develop its own innovation security strategy.

*The Cato Institute* is a libertarian think tank founded in 1977. The Institute’s reports show a strong and consistent commitment to libertarian principles. Its analysts recognize the increased role of the Chinese factor, but do not believe that China is such a serious threat as to justify the Trump administration’s abandonment of the free market in favor of protectionism. For them, the economic rise of China is indisputable; however, in their opinion, the provision about the effectiveness of the state capitalism model contains two critical errors. First, state capitalism has not been the driving force behind China’s past economic successes. On the contrary, these successes have been achieved thanks to Western investment and market reforms that China has been implementing since 1978. Second, China itself has faced systemic challenges that call into question whether it will stay on the same economic trajectory in the future [27].

Summing up the 50 years since the normalization of relations in 1972, the Institute’s analysts note that China poses a more serious challenge to US hegemony than the Soviet Union, but despite the scale, it is important not to exaggerate the threat. It consists only in the fact that China seeks to reproduce the American Monroe Doctrine. What is at stake is US influence in Asia, which is an important interest but not an existential threat to America’s future. In addition, the future of China is not fully defined either. Vulnerabilities include population aging, heavily indebted inefficient state-owned enterprises (China’s public debt is almost 300%), the bubble in the real estate market, and huge gaps in income. In the international arena, China has few real friends, not to mention allies; therefore, Trump made the mistake of declaring economic war on everyone, including US allies, instead of uniting with them against China [28].

The experts also emphasize that the confrontational position of the Biden administration towards China on issues of both trade and security is nothing more than a simplified version of Trump. The continuity of this policy is even more evident in the second case, especially with respect to Taiwan. The Biden administration continued the course and increased the US military presence in Taiwan. An ever-increasing role is played by the Congress, where a full bipartisan consensus has been reached. Legislative initiatives appear to reflect the intentions of both hawks and doves. A law is already being discussed that effectively gives the president carte blanche to defend Taiwan using US military forces without congressional approval [29].

*The Hudson Institute* is a conservative think tank founded in 1961. According to the analyst T. Duesterberg, the most important achievement of the Trump administration’s trade policy was the recognition that China is not a responsible actor despite many hopes. The White House has also become convinced that WTO rules and mechanisms are of little help in solving the Chinese problem. Although the actions of Trump caused discontent among the supporters of open trade, a number of countries (Australia, Japan) followed the US example. Duesterberg suggested that Biden would also focus on the restoration of industry and increased internal stability. Among the priorities, the return of the United States to the TPP was recommended, as well as the conclusion of FTAs with Taiwan and Britain. A good institutional structure that could serve as a platform for strengthening the coordination of defense production with allies is the Five Eyes group [30, p. 2], since among its political leaders there is an understanding that the Chinese strategy Made in China 2025 poses a serious threat to their competitiveness in high-tech sectors (especially AI) [31, p. 3]. In January 2022, Duesterberg outlined a number of acute problems hindering China’s economic growth that it had failed to solve: population aging, growing inequality, and degradation of the natural environment; the priority of state corporations, where nepotism and incompetence flourish at the expense of economic efficiency; media restriction; and reckless real estate schemes that increase the risk of a financial bubble.

As for security, in anticipation of NDS 2022, where China was cited as the number one strategic rival and challenge, analysts found that the United States had done too little to contain China in the South China Sea. China threatens its neighbors, and the United States must end this with a comprehensive strategy. Conducting continuous naval exercises was named as one of the instruments of pressure that would demonstrate the determination of the United States to protect its allies [32]. It should be clear that dissatisfaction with the actions of China is due to the creation of artificial, alluvial islands in the disputed waters of the Spratly archipelago and the Paracel Islands, and the deployment of military facilities on their territory. In July 2016, the Permanent Court of Arbitration ruled that China had no grounds for territorial claims. China did not recognize this decision.

*The Hoover Institution* is a conservative think tank, founded in 1919, and now part of the Stanford University system. In November 2018, a report was published on Chinese influence in the United States. It was noted that China’s turn to military–political rivalry with the United States has radically changed the nature of bilateral relations. For three and a half decades, China’s behavior on the world stage has been determined by the principles of “reform and opening up” and “peaceful coexistence.” Chinese leaders sought to emphasize that rapid economic development and great power status should not threaten either the existing world order or the interests of Asian neighbors. However, when Xi Jinping came to power in 2012, the situation changed. Under his leadership, Chinese politics acquired new features. It aims not only to redefine China’s place in the world, but also to promote the development model of an alternative Western model of liberal democracy. China is taking an increasingly aggressive and expansive stance on the world stage, and bilateral relations are becoming more hostile. The main topic of the report was “China’s interference in the internal affairs of the United States.” From the point of view of its authors, China intervenes ingeniously and decisively, which is why the economic and geostrategic losses of the United States have become increasingly significant, so the most effective defense is to strengthen democratic values and institutions [33].

In April 2022, a devastating article was published by the leading analyst of the Institute, L. Diamond. He moved away from the term *new cold war* but put it differently, noting that China is a neo-totalitarian superpower deeply hostile to democracy. China’s ultimate goal is global hegemony with dominance not only in world trade and finance but also in areas such as the Arctic, outer space, and international institutions, although primarily Chinese party leaders are obsessed with maintaining their 70-year monopoly on power. The challenge posed by the CCP is fundamental to US national security and values. Diamond pointed out that the “Chinese demonstration of hard power” is becoming increasingly audacious and gave quite strong arguments in support of his position. The response of the United States and its allies requires “an equally tough policy of constructive vigilance” based on transparency, reciprocity, and strengthening of their own institutions [34].

The existence of bipartisan support for the anti-Chinese course was also confirmed by experts from another think tank affiliated with the university environment—the Belfer Center, founded in 1973 at Harvard University [35, p. 2]. From their perspective, Washington had too much faith in its ability to determine China’s trajectory. China, on the other hand, was building up its power, increasing tension not only with the United States but also with its neighbors. The result has led many politicians in Washington to conclude that long-term efforts to create a constructive relationship with China integrated into the international system now look more ambitious than realistic. The United States must strengthen and develop relations with allies and partners by offering them an attractive alternative to China’s influence in the IPR. The intensive development of China represents the most serious geopolitical problem for the United States, and the challenge thrown by China will be more serious than the challenge from the Soviet Union.

## PERCEPTION OF THE CHINESE THREAT INSIDE THE US POLITICAL ELITE

Speaking frankly, the topic of the Chinese threat to the unipolar world order led by the United States was raised more than once even before Trump’s presidency. Under the Obama administration, Secretary of Defense E. Carter was very tough on China, noting that the United States and many other states are deeply concerned about some of the actions that China is taking, like the nontransparent defense budget, measures in cyberspace, and behavior in the South China and East China seas, which raise a number of serious questions [36]. The report of the US Senate Committee on Armed Services, published in May 2015, gave very harsh assessments of China’s actions in the South China Sea as expansionist territorial claims that unilaterally change the status quo and increase tension in the region [37].

Yet a significant shift in US–China relations took place under the Trump administration, and what made it unique was the speed with which it happened. It is known that Trump criticized China even during the 2016 election campaign. Having become president, Trump ensured that the sluggish discussion in the United States about containing China moved into the phase of concrete actions based on interparty consensus. In March 2018, when Trump imposed the first duties on Chinese goods, a trade war began between the United States and China, which led to the signing of an agreement on the first phase of the settlement of the trade dispute on January 15, 2020. For the United States, it turned out to be beneficial, as it managed not only to gain greater access to the Chinese domestic market, but also to weaken the position of its main strategic competitor. In November 2018, at the G20 summit in Argentina, the USMCA agreement was signed, which fits into the strategy to strengthen the US position in its “inner courtyard” and form a single anti-Chinese trade and economic cluster. Congressional acceptance of the USMCA agreement by an overwhelming majority and bipartisan support for tougher trade policy against China are evidence that the anti-China consensus in the United States is rapidly strengthening.

A growing number of dignitaries have begun to speak of a growing consensus within the establishment that sees China not only as a strategic challenge to the United States but also as a country that has risen at the expense of the United States and cannot be stopped by the tools of the global liberal world order. Political scientist E. Kordesman singled out five key speeches by White House employees that testify to a new confrontational approach to China: the speech of National Security Adviser R. O’Brien on June 24, 2020, in Phoenix at the opening of the TSMC microelectronics factory; the speech by FBI Director C. Wray at the Hudson Institute on July 7, 2020; the speech by Attorney General W. Barr on July 17, 2020 at the Gerald Ford Presidential Museum in Michigan; and two speeches by Secretary of State Mike Pompeo on July 23, 2020, at the Nixon Library in California and on July 13, 2020, at the State Department [38].

The arguments put forward by many influential voices in favor of the separation of the two countries have not gone anywhere. It will not be superfluous to give some excerpts. In October 2017, Secretary of State R. Tillerson said that the rules-based world order is increasingly under strain due to an authoritarian China that violates the sovereignty of other countries and whose provocative actions in the South China Sea directly defy international law and norms that are adhered to by the United States and other countries [39]. In October 2018, Vice President M. Pence made a very tough speech, emphasizing that China spends as much on its armed forces as the rest of Asia combined, and wants nothing less than to oust the United States from the Western Pacific [40]. In July 2020, Secretary of State M. Pompeo contrasted the “free world” with the “Marxist–Leninist regime” and shared the fears of R. Nixon, who considered that by opening the Chinese Communist Party to the world, he (Nixon) had created “Frankenstein.” Pompeo said, “Our policies—and those of other free nations—resurrected China’s failing economy only to see Beijing bite the international hands that were feeding it” [41].

There is no reason to believe that bilateral relations have returned to the status quo after Trump’s departure. Even during the 2020 summer debates, the main Democratic candidates (M. Bennett, P. Buttigieg, B. O’Rourke, T. Ryan, and E. Yang) showed a clearly confrontational approach to China. In September 2020, prominent neoconservative R. Kaplan noted that despite partisan polarization, both sides share deep concerns about China, and recalled that in February 2020 at the Munich Security Conference, House Speaker N. Pelosi noted, that they and Trump “have an agreement on the line of China.” According to Kaplan, unlike in previous years, China has few, if any, friends in the corridors of power in Washington. Even outside of Congress, there is a consensus emerging across the political spectrum about why China poses a threat to the United States and how to deal with it [42]. Thus, one gets the feeling that for the US political elite, China is at the forefront of a neo-authoritarian challenge that threatens the idea of liberal democracy. Competition with the United States unfolds in ideological terms and becomes a zero-sum game, and therefore the United States simply cannot give up its leadership, otherwise China will take its place.

The downward spiral in US–China relations has taken public opinion with it. Although Democrats criticize Trump’s confrontational approach to China and oppose the separation of the two economies, they agree that the United States should take a harder line on China, according to an October 2020 Chicago Global Affairs Council poll. Thus, for the first time in two decades, the majority of Americans (55%), Republicans (67%), independents (53%), and Democrats (47%) perceived China as a serious threat; favorable attitudes of Americans towards China have fallen to the lowest level (32 out of 100) in the history of polls since 1978; for cooperation and interaction with China, 47% (65% in 2006), while for the containment of China, 49% (29% in 2006); 64% of Republicans for opposition to China, 60% of Democrats for cooperation; the majority are in favor of building strong relationships with traditional allies (South Korea and Japan), even if this worsens relations with China (77% compared to 58% in 2010) [43].

In October 2020, a policy article by H. Clinton was published on the website of the *Foreign Affairs* magazine published by CFR [44]. The fact that it was written by one of the leaders of the Democratic Party gave it a special resonance. The following should be noted. First, almost everything in it corresponded to the directions set by Trump. Second, the article did not say much about Russia, but a lot was said about China, which is a completely new kind of asymmetric threat to the United States. Clinton stated that the declining industrial potential of the United States and insufficient investment in R&D make the country dangerously dependent on China and unprepared for future crises, while China is doing everything possible to increase its advantage. Clinton acknowledged that the strategic landscape had changed and urged Americans to adapt to it with the combination of two agendas: military modernization and internal renewal with the restoration of the country’s industrial and technological power. The fact that China is a threat was also recognized by J. Biden, who spent many hours with Chinese leaders and realized who the United States was dealing with. In his article, he stated that China is playing the long game by expanding its global presence, promoting its own political model, and investing in future technologies, and therefore the United States needs to maintain a tough relationship with China; after all, if it achieves its goal, it will continue to rob the United States and American companies [45].

This is the essence of Biden’s position: China poses a threat to the United States, which must be responded to. As he repeated more than once after the inauguration, the United States is facing a challenge to its prosperity, security, and democratic values from the most serious competitor—China [46]. In his first address to Congress, Biden attacked China, promising to maintain the US military presence in the IPR and accelerate the country’s technological development. Biden urged legislators to pass a comprehensive bipartisan package of laws to put pressure on Beijing considering its human rights violations, to eliminate trade imbalances, and to increase US R&D funding to compete more effectively [47]. In March 2022, the Senate passed the American Competition Act [48]. The multibillion-dollar law aims to maintain US industrial and technological dominance; concerns changing the supply chain and R&D to minimize dependence on Chinese-made products. It addresses issues of human rights, democracy, and the rule of law in China.

Against this backdrop, the Biden administration set about developing a comprehensive strategy for China, the general outlines of which can already be seen from the rhetoric and actions for 2021. The approach is based on the belief that China is moving in the wrong direction, but the possibilities to influence Beijing are limited, and so Washington needs to prepare for a long-term rivalry. In December 2021, in Jakarta, US Secretary of State E. Blinken presented the vision of a “free and open Indo–Pacific.” He noted that everyone in the region is concerned about “the aggressive actions of Beijing, which intends to turn the South China Sea into its own inland sea.” According to Blinken, this threatens the freedom of navigation and the movement of trade flows, so the United States will work with allies and partners to “protect the rule-based order” [49]. National Security Adviser J. Sullivan takes a softer rhetoric, arguing that the White House does not seek a fundamental transformation of the Chinese system and that the goal of US policy is to create conditions for the coexistence of two major powers in the international system.

In May 2022, Blinken delivered a speech outlining the White House’s approach to Beijing [50]. In accordance with it, the United States intends to protect and modernize the rules-based international order—a system of laws, agreements, principles, and institutions—that is undermined by China, declared the main long-term challenge to the international order. According to Blinken, the United States cannot rely on Beijing to change its policies to be more repressive at home and more aggressive abroad; it will rise to this challenge and advance its own vision of an open, inclusive international system.

It was clear from Blinken’s speech that Biden believes this decade will be pivotal, and, in order to succeed, his administration has developed a comprehensive three-pronged strategy: “invest, unite, compete.” The term *integrated deterrence* was also introduced to refer to a new approach based on the involvement of allies and partners; work in conventional, nuclear, space, and information fields; and building on US strengths in economics, technology, and diplomacy. The challenge from China will be a great test for American diplomacy, and therefore Blinken declared his determination to provide the State Department with all the necessary resources as part of the modernization program, including the creation of a “China House”—an integrated department-wide team—that will coordinate and implement US policy by working with Congress. It can be noted that this was one of the best performances on China in recent years. It is clear that there is a growing understanding in Washington of the need for a more realistic approach to relations with Beijing, largely in line with the recommendations that think tanks have been preparing for many years.

## CONCLUSIONS

The following conclusions suggest themselves. American think tanks play an important role in US policy making. Undoubtedly, one of the main trends of recent years is a fundamental change in their positions and approaches to China, which reflects a fundamental reassessment of how they understand the Chinese threat and recommend responding to it. Numerous reports indirectly confirm that a certain consensus on the issue of China has been formed among the think tanks of different ideological orientations. There was a shift in Beijing’s strategy to a more aggressive policy towards the West, aimed not only at redefining China’s place in the world but also at promoting the development model of an alternative Western model of liberal democracy. A telephone conversation between Biden and Xi in March 2022 confirmed concerns in this regard. As Biden said, the Chinese leader believes that an authoritarian regime is better in the new century since it does not require lengthy negotiations to develop the consensus needed in a liberal democratic system. Everyone unequivocally agrees on criticism of China’s inherent economic model. At the same time, several experts draw attention to the fact that China itself is facing systemic challenges that call into question whether it will stay on the same economic trajectory in the future.

For most analysts, the great power rivalry with China, which includes not only military and economic confrontation but also the struggle of two ideologies, is a long-term and historically unparalleled challenge to American national interests and values, to which the United States will have to adapt. It is recognized that China’s actions are ideologically motivated, and that the current Chinese ideology is fundamentally incompatible with American ideology; thus, the world is in a long-term strategic competition between the two systems, two models of the new world order. Almost all think tanks acknowledge that previous US administrations focused primarily on integrating China into the system based on the principles of the liberal world order, but this approach does not work. US efforts to integrate China into its world order, with the hope that it will become a “responsible actor” and accept the rules of the world order, have turned out to be a failure and have created threats to American leadership; thus, Washington needs a new grand strategy towards Beijing.

It would not be superfluous to note that the contribution of the Trump administration, which played an important role in awakening the United States to the growing Chinese threat to its hegemony, is recognized, but there are also shortcomings characteristic of the previous foreign policy course: inattention to alliances and institutions (withdrawal from the TPP), refusal from multilateralism, and, most importantly, the absence of a new grand strategy. Grand strategy is a question raised by absolutely all think tanks that have managed to develop appropriate recommendations for US government agencies. Thus, it is noted that the United States must reinvest in its unique strengths: in domestic policy, to solve economic problems and invest in factors of economic prosperity and national security; on a global scale, to return to a multilateral approach, to build and lead a single anti-Chinese bloc. The main struggle for world leadership will unfold in the IPR, which is becoming the main theater of great-power rivalry, where the intensification of relations with Taiwan, whose growing factor is noted by everyone, is of particular relevance.

Finally, and most importantly, judging by the statements and actions of the last two administrations, Trump’s and Biden’s, it becomes clear that the approaches of think tanks to China and recommendations to the US government, set out in numerous reports, influenced the positions of American politicians. Due to ideological differences between Democrats and Republicans, there is some friction over methods of and approaches to containing China; on how the government should position the United States in the face of authoritarian, communist China. However, all are unanimous on the main point: China, not Russia, is the main foreign policy problem for the United States; the time has come to move to a tougher line and, accumulating its power, in parallel to prepare for a long-term strategic rivalry.
